# Health and lifestyle of Nepalese migrants in the UK

**DOI:** 10.1186/1472-698X-8-6

**Published:** 2008-05-23

**Authors:** Pratik Adhikary, Padam P Simkhada, Edwin R van Teijlingen, Amalraj E Raja

**Affiliations:** 1 Department of Public Health, University of Aberdeen, Foresterhill Aberdeen AB25 2ZD, Scotland, UK

## Abstract

**Background:**

The health status and lifestyle of migrants is often poorer than that of the general population of their host countries. The Nepalese represent a relatively small, but growing, immigrant community in the UK, about whom very little is known in term of public health. Therefore, our study examined the health and lifestyle of Nepalese migrants in the UK.

**Methods:**

A cross-sectional survey of Nepalese migrants in UK was conducted in early 2007 using a postal, self-administered questionnaire in England and Scotland (n = 312), and telephone interviews in Wales (n = 15). The total response rate was 68% (327 out of 480). Data were analyzed to establish whether there are associations between socio-economic and lifestyle factors. A multivariate binary logistic regression was applied to find out independent effect of personal factors on health status.

**Results:**

The majority of respondents was male (75%), aged between 30 and 45 (66%), married or had a civil partner (83%), had university education (47%) and an annual family income (69%) ranging from £5,035 to £33,300. More than one third (39%) of the respondents have lived in the UK for 1 to 5 years and approximately half (46%) were longer-term residents. Most (95%) were registered with a family doctor, but only 38% with a dentist. A low proportion (14%) of respondents smoked but more than half (61%) consumed alcohol. More than half (57%) did not do regular exercises and nearly one fourth (23%) of respondents rated their health as poor. Self reported 'good' health status of the respondents was independently associated with immigration status and doing regular exercise

**Conclusion:**

The self reported health status and lifestyle, health seeking behaviour of Nepalese people who are residing in UK appears to be good. However, the overall regular exercise and dentist registration was rather poor. Health promotion, especially aimed at Nepalese migrants could help encourage them to exercise regularly and assist them to register with a dentist.

## Background

Migration is one of the key determinants of health and has a multiple, but complex, association. Health status of immigrants is a significant factor in their successful establishment in the host country. However, maintaining the optimum health and lifestyle of migrant populations has often been challenging both for migrants themselves and the host countries. The health of migrants, however, is also a function of the policies and practices that surround migration [1]. There are studies looking at migrants' health in UK [2-4]. Migrants' health depends on policies of host governments, legal status of migrants and the culture of both the sending and receiving the society [5]. The medical history of migrants, nature and quality of health care, social and health characteristics of re-settlement also determine the health of migrants [1].

Migrants from the Indian subcontinent had a higher death rate due to ischemic heart disease and cirrhosis of liver, but had fewer than expected deaths due to lung cancer and chronic bronchitis [6]. The high rate of mortality among South-Asian migrants in the UK from coronary heart disease, stroke and end-stage renal failure was also recently reported [7]. In addition, there was a high mortality among black African immigrants from stroke and end-stage renal failure [7]. However, mortality from coronary heart disease was lower, but stroke-related mortality was higher, among Caribbean migrants then the UK average. UK-born Caribbean and Irish men were more likely to be heavy smokers than the general population [8]. Elderly immigrants in UK, representing 6% of all seniors (aged 65 and over), were more vulnerable to depression. In addition, black immigrants had a higher rate of dementia because of uncontrolled hypertension [9,10]. Black Caribbeans, Black Africans, Indians, Pakistanis and Bangladeshis had more long-term illnesses than the white population, whilst the Chinese population had fewer of these problems [11]. Previous studies among South-Asians in England reported that they have poorer health status, poorer exercise and lower alcohol consumption than the general population [2-4]. Similarly, a study conducted among Ethiopian refugees in the UK found that many faced difficulties with the immigration status, unemployment, poor housing and access to social services, in part, due to their poor command of English. Moreover, many migrants, experienced difficulties accessing health services due to communication problems and poor understanding of the primary health care system that affected mental health and led to depression [12].

Until 2001, most Nepalese migrants went to India because of its open border. After 2001, the flow of people increased to other countries, especially Saudi Arabia (8.9%), Qatar (3.2%), the United Arab Emirates (1.7%) and Hong Kong (1.6%) [13]. In recent years, Nepalese emigration has increased all over the world [14]. The 2001 census of Nepal suggests that a total 7,221 Nepalese have settled in the UK [13]. However, this official number is an underestimate, as is estimated that about 100,000 Nepalese people are currently living in the UK including retired or currently serving Gurkha soldiers, businessmen, students and immigrants [15], whilst the Nepalese Embassy in London suggests a figure of 80,000 (personal communication). Migration of Nepalese people into the UK started initially with the recruitment of Gurkhas into the British Army. However, Nepalese migration to UK has significantly increased with the change in visa policy, e.g. highly skilled immigrants, of the UK Government [16]. A few studies have been conducted on ethnic minority populations in the UK. However, none have addressed the health, health-seeking behaviour and lifestyle of Nepalese migrants. So, the aim of this study was to examine health and lifestyle, health seeking behaviour of Nepalese people who are residing in UK. This paper provides basic information required for future planning and policy development to address public health issues in this population.

## Methods

A cross-sectional design was adopted for this questionnaire-based study, which was conducted in April-May 2007. As this study did not include research on NHS patients nor took place on NHS premises no ethical approval was needed from the Local Research Ethics Committee, as the university at which the authors are based does not have its own ethical review board no formal ethical approval could be obtained. The authors followed the ethical principles of the Helsinki Declaration [18]. The questionnaire was piloted before hand [19]. The study was both confidential and anonymous, respondents could refuse to take part in research and this was clearly stated on the questionnaire. Moreover, the researcher did not have access to the names and addresses of respondents and all questionnaires were returned anonymously.

A sample size calculation, assuming that 50% of the Nepalese migrants would report being in good health with an allowable variability of 5% and with 95% confidence, suggested we needed 380 subjects [20].

A total of 460 self completion questionnaires were distributed in England and Scotland; 312 replied. In addition, 20 people were approached for interviews in Wales and 15 respondents agreed to be interviewed. The total response rate was 68% (327 out of 480).

Migrants are a highly mobile population and it is hard to get actual number living in UK and it is more difficult to get numbers of Nepalese migrants living in different cities within UK. Therefore it is hard to get a sampling frame. To get a fairly representative sample of Nepalese migrants in UK, various steps were taken: (i) we contacted Nepalese community leaders in different cities to explore whether they maintained a list of Nepalese living in their area or asked about the possible community gatherings where large number of people could be met such as the Nepali New Year celebration; and (ii) contacts were made through Nepalese language media: such as a press-release in a local newspaper *Sandesh *(based in Peterborough), and a broadcast via the British Forces Broadcasting Services (BFBS) Gurkha radio to invite Nepalese to participate in the survey.

A structured questionnaire (designed both in Nepali and English languages) was used to collect information [see Additional file 1]. The questions covered socio-demographic information; self-assessed health status; health service use; attitudes towards being healthy; and life style factors (smoking, diet, drinking and regular exercise). The data were collected by distributing questionnaires to all individuals (one per family) attending the Nepali New Year celebration (15 April 2007) in London. The questionnaires were distributed to all the subscribers of a Nepali *Sandesh *newspaper. In addition, questionnaires were mailed to either individuals or community leaders to distribute it to Nepalese migrants living in that area. All questionnaires were accompanied by prepaid envelopes to increase the response rate (21). A few questionnaires were administered by telephone.

Data were entered in and analysed using SPSS version 14.0 (SPSS Inc., Chicago, IL, USA). Cross tabulations were generated between various demographic factors and lifestyle characteristics and health status. Chi-square test for trend was applied to find any association between ordinal categorical variable and binary outcome. Those factors which were significant in the univariate analysis were used in the multivariate analysis. Multivariate binary logistic regression was used to find out the best combination of factors associated with each outcome such as alcohol consumption, smoking habit, poor health status and poor/irregular exercise separately. A p-value of less than 0.05 was considered to be significant.

## Results

### Demographic and Socio-economic Characteristics

There were 327 Nepalese responded to the study. Most respondents were male. Two thirds were aged 30 to 45 with minimum of 18 years and maximum of 74 years. Most respondents were either married or had a civil partner. Nearly half of the respondents had a university degree, and another half had secondary/higher secondary level of education. A huge majority (84%) was resident of England and very few respondents (16%) lived in Scotland and Wales. Communities such as Brahmins/Chhetris comprised almost half of the respondents. The majority of respondents (69%) was in the middle-income range of £5,035 to 33,300 per annum. Two fifths of respondents were in UK between 1–5 years and nearly half of (46%) respondents mentioned their immigration status as Resident/Indefinite Leave to Remain/UK (Table 1).

**Table 1:** Demographic and socio-economic characteristics of the study subjects

**Variables (number of responses)**	**Number(n = 327)**	**Percentage**
**1. Age group**		
< 30 years	70	21.4
30–45	215	65.8
> 45 years	42	12.8
**2. Gender**		
Male	246	75.2
Female	81	24.8
**3. Marital status**		
Unmarried	55	16.8
Married/civil partner	270	82.6
Divorced/separated	2	0.6
**4. Education (n = 325)**		
None/primary	18	5.5
Secondary/higher secondary education	154	47.3
University	153	47.1
**5. Caste/ethnicity**		
Brahmin/chhetri	161	49.2
Gurung/tamang/sherpa	68	20.6
Rai/limbu	31	9.5
Newar	35	10.7
Magar	21	6.4
Other	11	3.4
**6. Place of residence in UK**		
England	276	84.4
Scotland	36	11.0
Wales	15	4.6
**7. Total family income (£) (per annum) (n = 279)**		
< 5035	52	18.6
5035–33,300	191	68.5
> 33,300	36	12.9
**8. Duration of stay in UK**		
< 1 year	40	12.2
(1–5) years	126	38.5
(6–10) years	89	27.2
> 10 years	72	22.0
**9. Immigration status (n = 317)**		
Resident, ILR/UK citizen	146	46.0
Work permit/fresh talent/highly skilled migrant/student/dependent/business	154	48.6
Refugee/asylum seeker/over stay	17	5.4
**10. Consumption of alcohol (n = 322)**		
Yes	196	60.9
No	126	39.1
**11. Tobacco (n = 324)**		
Yes	45	13.9
No	279	86.1
**12. Health Status (n = 326)**		
Poor	75	23.0
Good	251	77.0
**13. No Exercise (n = 326)**		
Yes	187	57.4
No	139	42.6

Only a small proportion of Nepalese migrants reported as suffering from chronic diseases such as diabetes (4.6%), high blood pressure (6.2%), high cholesterol (4.6%) and asthma (3.3%).

Most (77%) rated their health as 'good' whereas 23% rated their health as 'poor'. Almost all (95%) were registered with a GP (General Practitioner/family doctor) and only 38% respondents were registered with a dentist. Almost 80% respondents considered that they had a good diet. About two fifth of respondents did regular exercise. The health status of the respondents was statistically significantly associated with level of education and immigration status, whilst alcohol use was associated with age, gender, education and duration of stay (Table 2). Very few respondents (14%) were smokers. Interestingly, a much higher proportion (61%) consumed alcohol. Smoking was statistically significantly associated with gender only, and exercise was associated with education only (Table 2).

**Table 2:** Selected health and lifestyle status (Health Status, Tobacco Use, Alcohol Consumption, Exercise) and Demographic Characteristics.

**Demographic Factors (Response)**	**Health Status**	**Alcohol**	**Tobacco**	**No exercise**
	
	**(Poor)**	**(Yes)**	**(Yes)**	**(Yes)**
**Gender**				
Female	1	1	1	1
Male	1.58 (0.83–3.02)	10.86 (5.91–19.96)	3.9 (1.35–11.27)	0.84 (0.50–1.40)
**Age**				
> 45	1	1	1	1
30–45	0.75 (1.55–2.71)	0.89 (0.44–1.80)	1.41 (0.47–4.13)	1.70 (0.89–3.22)
< 30	0.50 (0.20–1.20)	0.32 (0.15–0.69)	1.86 (0.40–8.58)	1.54 (0.73–3.22)
	0.310	0.001	0.564	
**Education**				
None/primary	1	1	1	1
Secondary	0.25 (0.09–0.72)	1.87 (0.69–5.00)	0.12 (0.01–1.78)	0.14 (0.03–0.64)
Higher	0.28 (0.11–0.72)	2.10 (0.81–5.36)	0.40 (0.02–3.05)	0.16 (0.04–0.69)
**Duration of stay**				
< 1 year	1	1	1	1
1–5 year	1.14 (0.49–2.63)	0.46 (0.21–1.01)	1.40 (0.43–4.43)	1.17 (1.66–2.24)
6–10 year	1.58 (0.66–3.70)	3.00 (1.47–5.98)	1.18 (0.31–3.59)	1.66 (0.47–5.75)
> 10 year	0.96 (0.38–2.41)	2.34 (1.13–4.80)	2.17 (0.62–7.02)	1.57 (1.33–3.28)
				0
**Immigration Status in UK**				
Resident, ILR/UK Citizen	1	1	1	1
Work permit/fresh talent/highly skilled migrant/student/dependent/business	0.79 (0.41–1.50)	0.77 (0.52–1.13)	0.82 (0.42–1.59)	1.34 (0.90–1.97)
Refugees/asylum seeker/over stay	3.76 (1.30–10.69)	2.30 (0.64–8.16)	2.36 (0.67–8.16)	1.27 (0.44–3.52)

### Results of the logistic regression

Multivariate binary logistic regression models were constructed on the significant factors in univariate analysis for four outcomes of health and lifestyle such as alcohol consumption, smoking, health status, and regular exercise (Table 3). The socio-demographic factors along with associated life style factors: smoking, alcohol consumption and irregular exercise entered into the model were gender, age, education, ethnicity, immigration status, duration of stay and locality.

**Table 3:** Multivariate Logistic regression model for different health and lifestyle outcomes

Variable	OR	95% CI
**Model 1 – Alcohol Consumption**		
Gender Male	9.7	5.1–18.3
Age 45 + years	1.0	
30–45 years	1.0	0.4–2.3
< 30 years	0.4	0.1–0.9
Smoking	7.2	2.3–22.4
		
**Model 2 – Smoking Habit**		
Drunkeness	7.4	2.6–21.4
		
**Model 3 – Poor Health status**		
Immigration status in UK		
Resident, ILR/UK Citizen	1.0	
Work permit/Fresh Talent/Highly Skilled Migrant/Student/Dependent	0.9	0.5–1.7
Refugees/Asylum seeker/Over stay	5.2	1.5–17.6
irregular Exercise	2.9	1.5–5.6
		
**Model 4 **– **Irregular Exercise**		
Education		
None/Primary	1.0	
Secondary/SLC	0.1	0.03–0.7
Higher Secondary/University	0.2	0.05–1.1

Gender, age and smoking were significantly associated with alcohol consumption in multivariate analysis. Males were ten times more likely to be alcohol drinkers than females (OR = 9.7, 95% CI = 5.1 to 18.3). Similarly individuals who were between 30–45 years were equally likely to drink as individuals above 45 years, whereas the individuals with the age less than 30 years keep away from alcohol consumption (OR = 0.4, 95% CI = 0.1 to 0.9).

Alcohol consumption was significantly associated with smoking in the multivariate analysis. Alcohol drinkers were seven times more likely to be smokers than non-drinkers (OR = 7.4, 95% CI = 2.6 to 21.4).

Immigration status and regular exercise were significantly associated with poor health status in the multivariate analysis. Individuals who were refugee/asylum seeker/stay over 10 years in the UK were 5 times more (OR = 5.2, 95% CI = 1.5 to 17.7) likely to be having poor health status than individuals who have UK residentship. Similarly, individuals who were doing irregular exercise were three times more (OR = 2.9; 95% CI = 1.5 to 5.6) likely to have poor health status than individuals who did regular exercise.

Education was significantly associated with poor/irregular exercise in the multivariate analysis. Individuals who did secondary/SLC (school leaving certificate) level of education were likely to do more exercise (OR = 0.1; 95% CI = 0.03 to 0.7) than none/primary level of education.

## Discussion

The majority of respondents was male (75%) and aged between 30 and 45. This is due to the age distribution in this immigrant group, which is rather natural given the fact that immigrant groups are often dominated by young and middle-aged men. There are more male emigrants (83%) than female (17%) from Nepal [13]. Young and middle-aged men are more likely to migrate for work and further education [22].

The self-reported health status of 'poor' among Nepalese migrants in UK (23% overall, 25% for males and 17% for females) is comparable to that reported for the general population in England (23% for males and 26% for females) [2] and Scotland (25% overall) [23]. However, the percentage of Nepalese migrants reporting 'poor' health status is lower than that reported for other ethnic migrants in England (32% for men and 35% for women among Bangladeshi, 31% for men and 30% for women among Indians, 29% for men and 21% for women among Irish, 27% for men and 35% for women among Pakistani, and 26% for men and 38% for women among Black Caribbeans) [2]. The 'poor' health status reported by the Nepalese migrants in UK in this study is comparatively better than that reported by Nepalese migrants in the US where 42% rated their health as 'poor' [24].

Only a small proportion of Nepalese migrants reported suffering from chronic diseases such as diabetes (4.6%), high blood pressure (6.2%), high cholesterol (4.6%) and asthma (3.3%). This is significantly lower than that reported for the general population (24.8%) of Scotland [23]. This could be due to age effects, i.e. most respondents are young and middle-aged group or educational effects. Health status was significantly associated with the level of education. As most Nepalese migrants in this study were highly educated, this could have been one of the reasons why the prevalence of chronic diseases was lower among Nepalese migrants in UK.

Health status in this study is significantly associated with immigration status and regular exercise. People, with immigration status as refugees, asylum seeker and overstay were more likely to report 'poor' health than people with other immigration statuses (e.g. work permit/fresh talent/highly skilled migrant/student dependent/business). Immigrants who had not done regular exercise were more likely to report 'poor' health than those who had done so.

This study demonstrated that Nepalese migrants were not regularly involved in exercise. More than half (overall 57%, 56% for males and 61% for females) of Nepalese migrants reported that they do not regularly exercise. This is much higher than the proportion of general population of England not involved in regular exercise (21% men and 25% women) [4]. However, the proportion of Nepalese migrants in UK not involved in regular exercise was similar to the proportion of other ethnic migrant groups with poor or no regular exercises. The English Health Survey found that Indian, Pakistani, Bangladeshi and Black African men and women were less likely to participate in sports and exercise than men and women in the general population [4].

In this study, the level of education was associated with poor/irregular exercise. Respondents having higher education (e.g., university degree) were less likely not to be involved in regular exercises. It was, however, surprising that higher proportions of some of the highly educated group (e.g., health care workers) were also not involved in regular exercise. However, automation (e.g. the introduction of labour saving devices) and long working hours often associated with these professions might have a negative influence on their involvement in regular exercise [25].

This study identified an overall low smoking rate (14% overall, 17% for males and 5% for females) among Nepalese migrants in UK. This is significantly lower than that recorded for other migrant communities in England [22]. The English survey reported the smoking rates for minority ethnic groups as 40% for men and 2% for women among Bangladeshi, 30% for men and 26% for women among Irish, 29% for men and 5% for women among Pakistani, 25% for men and 24% for women among Black Caribbeans, 21% for men and 10% for women among Black African, 21% for men and 8% for women among Chinese, and 20% for men and 5% for women among Indian groups. Smoking rates in the general population of England were 24% for men and 23% for women [26].

The self-reported smoking rate in the current study among Nepalese migrants in UK (e.g., 17% among people < 30 years) is also significantly lower than the reported smoking rate of Nepalese living in Nepal (> 70% for age below 30 years) [27]. The female smoking rate among women is considered to be one of the highest in the world, especially among women living in the rural areas of Nepal. Some studies, e.g. a global health professional survey, indicated it to be as high as 73% among certain ethnic groups [27] which is considerably higher than the 5% recorded among women in our study. The high smoking rate recorded among women in rural Nepal might have been due to their low level of education. Furthermore, poverty, culture, tradition, environment, and family background are other factors that might have affected smoking rates. In contrast to people living in rural Nepal, Nepalese migrants to UK are highly educated.

The overall alcohol consumption rate of 61% in the present study (74% for males and 21% for females) was higher than the overall alcohol consumption in Nepal (male 33.6%; female 15.3% and average 23.6%) [28]. However, our study reported lower levels than that in the English general population. The 2004 Health Survey for England recorded alcohol users as those who drank at least once every 2 months, for the total population in England the rates were 87% for men and 76% for women, for other minority ethnic immigrant groups it recorded 87% for men and 83% for women among the Irish, 69% for men and 48% for women among the Chinese, and 79% for men and 65% for women among Black Caribbeans [3]. However, it has to be noted that other South Asian immigrant groups in England also have a low rates of alcohol consumption (3% for men and 2% for women among Bangladeshi and 11% for men and 5% for women among Pakistani [3]. Overall, alcohol consumption in our study was associated with the gender, age, education, smoking and the duration of stay in UK. Relatively more people (68%) in the older age group (> 45 years) were more likely to consume alcohol than the younger age group (41%) and alcohol consumption increased with the increase in the duration of stay in UK.

Results from the logistic regression showed that alcohol consumption is significantly associated with gender, smoking habit and age. The reason for higher consumption of alcohol among smokers may be due to the cultural practices or personal preference or beliefs on the health and lifestyle. For example, someone who is health conscious is less likely to smoke or consume too much alcohol. This study did not investigate possible reasons for higher drinking rates in males than the females, although this trend seems to be universal among all ethnic groups and also among the general population in England [3]. It is likely that males are more adventurous or less attentive about their own health, and therefore consume alcohol more frequently. Alternately, it could be that males often being the bread winner for the family, especially among the ethnic immigrants, drink more to relieve the burden of economic or physical hardship.

This is the first health and lifestyle study of Nepalese migrants in UK and provides some valuable insights into their health and behaviour. However, there are number of limitations in this study. Due to the time and resource constraint, and the absence of a complete list of Nepalese migrants in UK (i.e. a sampling frame), it was impossible to conduct a randomized study. Moreover, migrants are mobile population and it was difficult to identify their postal addresses. As a result, the study could not cover all areas of the UK. Hence, the survey depended on the self completion of the questionnaire by the study participants.

Respondents were more likely to be male than female, more of Brahmin/Chhetri ethnic groups and more university graduates than others. Hence, the study might not be representative of all possible groups, as illegal or semi-legal immigrants are less likely to participate in research This ties in with the finding that assuring anonymity in surveys appears to have a negative effect on response rates on 'population in flux' [29].

The response rate obtained in the survey was moderate. However, as the detailed information on individuals to whom the questionnaire was distributed was not available, it was not possible to identify the characteristics of non responders. So, the results of this study will have limited external validity and should be extrapolated with caution. Mental health is an important area for study among migrants as they often suffer from psychological stress in the new environment, but was not covered in this study.

## Conclusion

Health status (self reported) of Nepalese immigrants is better than the UK population and many of the immigrant populations from Asia. Overall, involvement of Nepalese migrants in regular exercise and their registration with a dentist was poorer than that reported for other UK minority ethnic groups. However, almost all respondents in this study were registered with a GP. Education was the major determinants of regular exercise which in turn, together with the immigration status, was the determining factor for good health status. As people with better education were likely to have a better job and healthy lifestyle, they had overall better health status than other immigrants. Settlement policies on the other hand, especially for the Nepalese migrants, should focus on encouraging them to exercise regularly and assist them to register with dentist.

## Competing interests

The authors declare that they have no competing interests.

## Authors' contributions

PA, PPS and ERvT were involved in the conception and design of the study. PA carried out the data collection. PPS and ERvT supervised the data collection and PA conducted statistical analysis under supervision of AER. PA drafted the paper with contributions from the co-authors. All authors read and approved the final manuscript.

## Pre-publication history

The pre-publication history for this paper can be accessed here:



## Supplementary Material

Additional file 1 Survey of Health and Lifestyle of Nepalese Migrants in the UK. Click here for file
