# Supplementary material for: Health and lifestyle of Nepalese migrants in the UK
Source: BMC Int Health Hum Rights. 2008 May 23;8:6. doi: 10.1186/1472-698X-8-6 (PMC2432045; doi:10.1186/1472-698X-8-6)
Supplement: Additional file 1 — Survey of Health and Lifestyle of Nepalese Migrants in the UK. [file 1472-698X-8-6-S1.doc]

**The University of Aberdeen, Department of Public Health**

**Aberdeen, AB25 2ZD**

**Survey of Health and Lifestyle of Nepalese Migrants in the UK**

**संयुक्त अधिराज्य (UK) मा बासस्थान भएका नेपालीहरुको स्वास्थ्य र जीवन शैलीको सर्वेक्षण**

आजको मिति (Today’s date): गते(Day): ……./ महिना(Month) ………………../2007

म प्रतिक अधिकारी एबर्डीन विश्वविद्यालयमा अध्ययन गर्ने विद्यार्थी हुँ। मैले UK मा वासस्थान भएका नेपालीहरूको **स्वास्थ्य र जीवन शैली संबन्धि अध्ययन गर्दै छु। तपाईंको अमूल्य समयको केहि मिनेट दिई यो तलको सर्वेक्षणका प्रश्नहरुको जवाफ दिनुहुन म हार्दिक अनुरोध गर्दछु। जवाफ बेनामी हुनेछन् र यसमा भएका संपूर्ण जानकारी अति गोप्य रहनेछन्। भरेको प्रश्नपत्र तल दिईएको ठेगानामा पठाईदिनु होला।**

**(**I am Pratik Adhikary, a Nepalese student at the University of Aberdeen and I am conducting a survey on the health and life-style of Nepalese migrants in the UK. It would be greatly appreciated if you could take a few minutes to fill out the questionnaire. Results are anonymous (I am not asking your name), confidential and for this study only. Please return your completed questionnaire to the address below.)

यदि तपाईंले यी प्रश्नहरुको जवाफ हालसालै पठाईसक्नु भएको भए, यो प्रश्नपत्र पुन: भर्नु पर्दैन। (Have you filled in this questionnaire before? If yes, thank you and stop. If no, please complete the following questions.)

**Demographic characteristics:**

1. तपाईंको लिङ्ग Are you (please tick one): पुरुष(Male): 🗖1 महिला(Female): 🗖2
2. तपाईंको उमेर (What is your age)? ________ (बर्षमा, completed years)
3. नेपालमा तपाईंको जन्मस्थान (जिल्ला) (Where were you born in Nepal? please specify the district name) ____________
4. तपाईं UK मा कुन शहरमा बस्नुहुन्छ (Where do you live in the UK)? (please specify city/town) ___________________
5. तपाईंको जातीय समूह (एउटा छान्नुहोस्) (What is your ethnic background?, please tick one)

| बाहुन / क्षत्री (Bramin/Chhetri) | 🗖1 | नेवार (Newar) | 🗖5 |
| --- | --- | --- | --- |
| गुरुङ (Gurung) | 🗖2 | मगर (Magar) | 🗖6 |
| तामाङ (Tamang) | 🗖3 | शेर्पा (Sherpa) | 🗖7 |
| राई (Rai) | 🗖4 | लिम्बु (Limbu) | 🗖8 |
| अरु (खुलाउनुहोस्) Other (specify): ___________________________ | | | |

1. तपाईंको वैवाहिक अवस्था (एउटा छान्नुहोस्) (What is your marital status?) (please tick one)

| अविवाहित (Unmarried) | 🗖1 | बिधुर/बिधवा (Widowed) | 🗖3 |
| --- | --- | --- | --- |
| विवाहित (Married/Civil partner) | 🗖2 | संबन्ध बिच्छेद । छुट्टै बसेको (Divorced/separated) | 🗖4 |

1. तपाईंका आफैंले हेरचाह गर्नुपर्ने बालबच्चा छन्? (Do you have dependent children?)

छन् (Yes) 🗖1 छैनन् (No) 🗖2

1. तपाईंसंग UK मा कति बच्चाहरू संगै बस्छन्? (How many of children live with you in UK?) __________। कतिजना बच्चाहरु नेपालमा छन्? (How many children live in Nepal?) __________
2. तपाईंको UK को बासस्थानमा कति कोठाहरु छन्? (How many bedrooms are at your UK residence ?) (house/apartment)? __________
3. तपाईंले (तपाईं र तपाईंको परिवारले) कति कोठाहरु प्रयोग गर्नु भएको छ? (How many bedrooms do you (=you, partner & children together) use?) ___________
4. तपाईं बस्नुभएको घर भाडामा हो कि आफ्नै हो? (Are you the tenant or the owner of the house/apartment where you live?)

भाडामा (Tenant): 🗖1 आफ्नै घर (Owner): 🗖2

1. तपाईं सुरुमा UK कसरी आउनु भएको थियो? (In what capacity did you first come to the UK (please tick one)?

| पर्यटक भिसामा (As a visitor) | 🗖1 | कार्य अनुमति (वर्क पर्मिट) (As a work permit holder) | 🗖4 |
| --- | --- | --- | --- |
| विद्यार्थी (As a student) | 🗖2 | उच्चदक्ष जनशक्ती (As a highly skilled migrant) | 🗖5 |
| परिवारसंग (As a dependant) | 🗖3 | ब्रीटीस सेना (As an Ex-Gurkha, British army) | 🗖6 |
| अन्य (खुलाउनुहोस) Other (specify) | | _____________________________ |  |

1. तपाईं UK मा बस्नुभएको कति बर्ष भयो? (How long have you been in UK? number years & months) _______ (बर्ष, years) & ______ (महिना, months)
2. तपाईंको UK को भिसा / बसाई कस्तो हो? (What is your current immigration status in the UK?, please tick only one)

| स्थायी बासिन्दा (Resident, ILR) | 🗖1 | नागरिक (UK citizen) | 🗖6 |
| --- | --- | --- | --- |
| कार्य अनुमती (Work permit) | 🗖2 | विद्यार्थी (On student visa) | 🗖7 |
| व्यापारिक (Business status) | 🗖3 | परिवारका साथ (Dependant) | 🗖8 |
| शरणार्थी (Refugee/asylum seeker) | 🗖4 | लुकेर बसेको (Over stay) | 🗖9 |
| फ्रेस टेलेन्ट (Fresh Talent) | 🗖5 | उच्चदक्ष बसाईंसरुवा (Highly Skilled Migrant) | 🗖10 |
| अन्य (खुलाउनुहोस) Other (specify) | _____________________________ | |  |

1. तपाईंको UK मा मुख्य पेशा (What is your primary occupation in the UK?) (please tick one only)

| **cWoog**  (Study) | 🗖1 | घरेलु सहायक (Domestic Assistant) | 🗖7 |
| --- | --- | --- | --- |
| **lrlsT;s / g;{** (Doctor/Nurse) | 🗖2 | स्वास्थ्य सहायक (Care Assistant) | 🗖8 |
| Manager/Account/Administrator  -d]g]h/ / n]vfkfn / k|zf;s_ | 🗖3 | सेफ/वेटर (रेष्टुरेन्ट) Restaurant (chef/wait | 🗖9 |
| Engineering/IT specialist  **-O{GhLlgo/ / ;"rgf k|aLlw laz]if1_** | 🗖4 | सुरक्षा गार्ड (Security) 🗖10 | |
| Teacher/Researcher/Lecturer  **-lzIfs / cg;Gwfgstf{ / k|fWofks** | 🗖5 | अन्य (खुलाउनुहोस) (Other, specify) _______________ | |
| व्यापार (Business) | 🗖6 |  | |

1. तपाईंले कति पढाइ पुरा गर्नु भएको छ? (Please indicate the highest level of formal education you have completed, tick one only)

| अशिक्षित (None) | 🗖1 | कलेज तह (I.A/I.Ed/ ISc /10+2) | 🗖4 |
| --- | --- | --- | --- |
| प्राथमिक तह (Primary) | 🗖2 | विश्वविद्यालय तह (University) | 🗖5 |
| माध्यमिक तह (Secondary/SLC pass) | 🗖3 |  |  |

1. तपाईं आफ्नो अंग्रेजीलाई कुन स्तरको ठान्नु हुन्छ? (How do you rate your English proficiency?, please tick one)?

| अति राम्रो (Very good) | 🗖1 | कमजोर (Poor) | 🗖4 |
| --- | --- | --- | --- |
| राम्रो (Good) | 🗖2 | धेरै कमजोर (Very Poor) | 🗖5 |
| ठिकै (Fair) | 🗖3 |  |  |

1. तपाईंको परिवारको सन् २००६ मा जम्मा बार्षिक आम्दानी (कर तिर्नु भन्दा पहिले) कति थियो? (What was your total family income before taxes for 2006? (for single, indicate your personal income). ₤___________

**स्वास्थ्य र जीवन शैली (General Health and Lifestyle):**

1. सामान्यतया, तपाईंले आफ्नो स्वास्थ्य कस्तो छ भन्ने ठान्नु हुन्छ? (In general, how would you rate your health?, please tick one).

| अति राम्रो (Very good) | 🗖1 | नराम्रो (Poor) | 🗖4 |
| --- | --- | --- | --- |
| राम्रो (Good) | 🗖2 | धेरै नराम्रो (Very Poor) | 🗖5 |
| ठिकै (Fair) | 🗖3 |  |  |

1. सामान्यतया, तपाईंले आफ्नो खानपीन कत्तिको स्वस्थ्यकर छ भन्ने ठान्नु हुन्छ? (In general, how healthy would you consider your diet?, please tick one).

| अति स्वस्थ (Very good) | 🗖1 | अस्वस्थ (Poor) | 🗖4 |
| --- | --- | --- | --- |
| स्वस्थ (Good) | 🗖2 | धेरै अस्वस्थ (Very Poor) | 🗖5 |
| ठिकै (Fair) | 🗖3 |  |  |

1. के तपाईं धुम्रपान गर्नुहुन्छ? (Do you smoke?)

गर्छु (Yes): 🗖1 गर्दिन (No) : 🗖2 (नगर्ने भए, २३ मा जानुस्, If no, go to 23).

धुम्रपान गर्ने भए एक दिनमा कतिवटा चुरोट पिउनु हुन्छ? (If yes, how many cigarettes do you smoke during an average day?) ________

1. तपाईंको बिचारमा, के तपाईंले पिउने चुरोटको संख्या तपाईंको स्वास्थ्यको लागि हानिकारक हुन्छहोला? (Do you think your present level of tobacco consumption is harmful to your health?)

हानिकारक छ (Yes): 🗖1 हानिकारक छैन (No): 🗖 2 थाहा छैन (Not sure): 🗖 3

1. के तपाईं मादकपदार्थ (रक्सी, बियर आदि) सेवन गर्नुहुन्छ? (Do you drink alcohol?)

गर्छु (Yes): 🗖1 गर्दिन (No): 🗖2 (नगर्ने भए, २६ मा जानुस् , If no go to 26).

1. तपाईं कतिको मादकपदार्थ सेवन गर्नुहुन्छ ? (How often do you drink alcohol?

| **(b}lgs)** Daily | 🗖1 | (**xKtfdf ! k6s**) Once a week | 🗖4 |
| --- | --- | --- | --- |
| **(k|fo b}lgs)** Almost daily | 🗖2 | **(slxn]sfxL)** Occasionally/Only in the function | 🗖5 |
| (**xKtfdf @/# k6s**) 2-3 times per week | 🗖3 |  |  |

1. तपाईंको बिचारमा के तपाईंले पिउने मादकपदार्थको मात्रा स्वास्थ्यको लागि हानिकारक हुन्छ होला? (Do you think your present level of alcohol consumption is harmful to your health?)

हुन्छ (Yes): 🗖1 हुंदैन (No): 🗖2 थाहा छैन (Not sure): 🗖3

1. तपाईंको लागि एक हप्तामा कति ईकाइसम्म मादकपदार्थ सेवन गर्न उचित होला? How many units of alcohol consumption is considered safe and sensible for you in a week?)

_______ ईकाइ (units) थाहा छैन (Do not know): 🗖 9

1. तपाईं फलफूल र सागसब्जी कति खानुहुन्छ? (फलफूल र सागसब्जीको लागि छुट्टा छुट्टै उत्तर दिनु होस्) (How often do you eat fruits and vegetables/Salad (include vegetables in curry)? Please tick one box for vegetables AND one box for fruit)

| **सागसब्जी, ;nfb (Vegetables and salad)** | | **फलफूल (Fruits)** | |
| --- | --- | --- | --- |
| दिनको ३ पटक भन्दा बढी (More than 3 times a day) | 🗖1 | दिनको ३ j6f भन्दा बढी (More than 3 times a day) | 🗖1 |
| दिनको १ पटक भन्दा बढी (More than once a day) | 🗖2 | दिनको १ j6f भन्दा बढी (More than once a day) | 🗖2 |
| दिनको १ पटक (One time a day) | 🗖3 | दिनको १ j6f (One time a day) | 🗖3 |
| दिनको १ पटक भन्दा कम (Less than once a day) | 🗖4 | दिनको १ j6f भन्दा कम (Less than once a day) | 🗖4 |
| हप्ताको १ पटक भन्दा कम (Less than once a week) | 🗖5 | हप्ताको १ j6f भन्दा कम (Less than once a week) | 🗖5 |

1. के तपाईं नियमित रुपमा कसरथ/व्यायाम गर्नुहुन्छ? (Do you exercise regularly?)

गर्छु (Yes): 🗖1 गर्दिन (No): 🗖2

गर्ने भए के गर्नु हुन्छ? (If yes, what do you do regularly?)

हिड्छु (Walk): 🗖1 जीम जान्छु (Go to the gym): 🗖3

खेल खल्छु (Play sports) 🗖2 आँफै कसरथ गर्छु (Self exercise): 🗖4

1. के तपाईंको आफ्नो GP छ वा GP को मा दर्ता हुनु भएको छ? (Are you registered with a General Practitioner (GP)?)

छ (Yes): 🗖1 छैन (No):2 🗖 (छैन भने, ३१ मा जानुस्, If no, go to question 31)

1. गत १२ महिनामा, तपाईं आफ्नोलागि कति पटक GP को मा जानु भयो? (Approximately how many times in the last 12 months did you visit a GP in UK?) ______(पटक, times)
2. GP संग दर्ता नभएको भए, किन दर्ता नगर्नु भएको हो? (If no, why were not you registered with a GP?, tick most appropriate).

_____________________________________________________________

32. के तपाईंले गत १२ महिनामा स्वास्थ्य परिक्षण (रोग नलागिकनै) गराउनु भयो? (Have you had a general medical check up in the last 12 months (without being sick)?)

गराएँ (Yes): 🗖 1 गराईन (No): 🗖 2

33. के तपाईंले विगत ३ बर्षमा कुनै रोगको स्क्रिनिङ टेस्ट (जस्तै, स्तनको मेमोग्राम, प्याप टेस्ट, कोलेस्टेरोल टेस्ट, प्रोस्टेट टेस्ट आदि) गर्नु भएको थियो? (Have you gone for a disease screening test in the last 3 years? (e.g., mammogram, PAP test, blood cholesterol monitoring, prostrate test)

थिएँ (Yes):🗖 1 थिएन (No):🗖 2

34. तपाईंलाई गत १२ महिनामा कति पटक अस्पतालको दुर्घटना तथा आकस्मिक विभागमा जानु पर्‌यो? How many times in the last 12 months did you visit an Accident & Emergency Department?

______ (पटक, times)

35. तपाईंको आफ्नो दन्त चिकित्सक छ वा तपाईं दन्त चिकित्सककहाँ दर्ता हुनुभएको छ? (Are you registered with a dentist?)

छ (Yes): 🗖 1 छैन (No): 🗖 2 (छैन भने ३७ मा जानुस्, If no, go to question 36)

36. तपाईं गत वर्ष कति पटक दन्त चिकित्सक कहाँ जानु भयो? (Approximately how many times in the last 12 months did you visit your dentist in UK?) _____(पटक, times)

37. के तपाईं दीर्घ रोघबाट पिडित हुनुहुन्छ? (मिल्ने जति सवैमा रेजा लगाउनुहोस्) (Do you have any chronic health problems/conditions?, tick all that apply):

A. मधुमेह (Diabetes) Yes: 🗖1 No 🗖2

B. उच्च रक्तचाप (High blood pressure) Yes: 🗖1 No 🗖2

C. उच्च कोलेस्टेरोल (High cholesterol) Yes: 🗖1 No 🗖2

D. दम (Asthma) Yes: 🗖1 No 🗖2

E. अन्य (Others, specify): _______________________

38 स्वास्थ्य र रोग संबन्धि तपाईको धारणा/विचार (प्रत्येक हरफमा एक चिन्ह लगाउनुहोस्) (Your opinions on health and illness, tick one answer on each line /sentence please)

| SN | भनाई (Statements) | पूर्ण सहमत (Strongly Agree)  1 | सहमत (Agree)  2 | थाहा छैन / अरुमा कुरामा भर पर्छ (All depends / Don’t know)  3 | असहमत (Disagree)  4 | पूर्ण असहमत (Strongly disagree)  5 |
| --- | --- | --- | --- | --- | --- | --- |
| a | डाक्टरको सल्लाह अनुसार गर्नु ठिक हुन्छ (It’s sensible to do exactly what the doctors advise) |  |  |  |  |  |
| b | राम्रो स्वास्थ्य हुनु भनेको जीवनको सबैभन्दा ठूलो कुरो हो (To have good health is the most important thing in life) |  |  |  |  |  |
| c | स्वास्थ्य भनेको भाग्यको कुरो हो (Generally health is a matter of luck) |  |  |  |  |  |
| d | स्वास्थ्यको बारेमा जति धेरै सोच्यो त्यतिनै धेरै बिरामी परिन्छ (If you think too much about your health, you are more likely to be ill) |  |  |  |  |  |
| e | रोग लाग्नु भनेको दैवको खेल हो (Suffering sometimes has a divine purpose) |  |  |  |  |  |
| f | सिकिस्त बिरामि नभैकन म डाक्टरकोमा जान्न (I have to be very ill before I’ll go to the doctor) |  |  |  |  |  |
| g | मान्छेलाई स्वास्थ्यको बारेमा सोच्ने फुर्सदै कहाँ छ (People don’t really have time to think about their health) |  |  |  |  |  |

39. बिरामी हुनुभयो भने, तपाईं पहिले के गर्नुहुन्छ? (सबै भन्दा पहिले गर्ने कुरालाई १, दोश्रो गर्नेलाई २, गर्दै नंबर दिनुहोस्) (If you get ill, what would you do first?, prioritize: 1 for the thing that you will do first, 2 for the second option, 3 for 3^rd^ option and so on)

|  | प्राथमिकता Priority (1 – 5) |
| --- | --- |
| साथी वा परिवारको मान्छेसंग उपचारको सल्लाह माग्छु (Ask a friend or family for medical advice) | _____ |
| आँफै औषधी (डाक्टरी दवाई) खान्छु (Self medication / using western medicine) | _____ |
| डाक्टरी भन्दा अन्य उपचार (आयुर्वेदिक, अकुपङ्चर, चिनिया उपचार आदि) गराउँछु (Use Alternative medicine (Ayurvedic, acupuncture, Chinese medicine etc.)) | _____ |
| डाक्टरकहाँ जान्छु (See a physician / doctor) | _____ |
| औषधी पसले (फार्मासिस्ट) संग सल्लाह लिन्छु (See a pharmacist) | _____ |

40 तपाईंलाई UK मा लाग्ने मुख्य समस्या/चिन्ताको विषय के हो? (सबभन्दा मुख्यमा चिन्ह लगाउनुस्) (What is your main concern/worry in the UK?, tick most important one only).

| सामाजीक सहयोगको अभाव (Lack of social support) | 🗖 1 |
| --- | --- |
| आर्थीक समस्या/कठिनाइ (Economic hardship) | 🗖 2 |
| मेसिन जस्तो काम गर्नु पर्ने, आफ्नो परंपरागत जीवन शैली हराउँदै गयो (Mechanistic lifestyle and loss of traditional living) | 🗖 3 |
| मौसम (Climate) | 🗖 4 |
| आँफुलाई मनपर्ने खानेकुरा नपाइनु र चाडबाड भनेजस्तो मनाउन नपाउनु (Lack of traditional food and celebrations) | 🗖 5 |
| अध्यागमन/भीसाको समस्या (Immigration status/Visa status) | 🗖 6 |
| अन्य (खुलाउनुस्) Other specify: …………………………… | 🗖 |

41 के तपाईं नेपाली समाज, संगठन, सांस्क्रीतिक समूह आदिसंग संबन्धित हुनुहुन्छ? (Are you associated with any communities (social organization/ cultural associations/ etc.) made up by people from your country?)

छु (Yes): 🗖 1 छैन (No): 🗖 2

तपाईंको अमूल्य सहयोगका लागि धेरै धेरै धन्यवाद। क्रिपया यसलाई मेरो ठेगानामा **htL ;Sbf] l56f**] पठाईदिनु होला। (Thank you very much for your support and help. Please return the completed questionnaire to my address as soon as possible:

**Pratik Adhikary,**

**Dept. of Public Health,**

**University of Aberdeen,**

**Aberdeen, AB25 2ZD**

**Scotland, UK**
